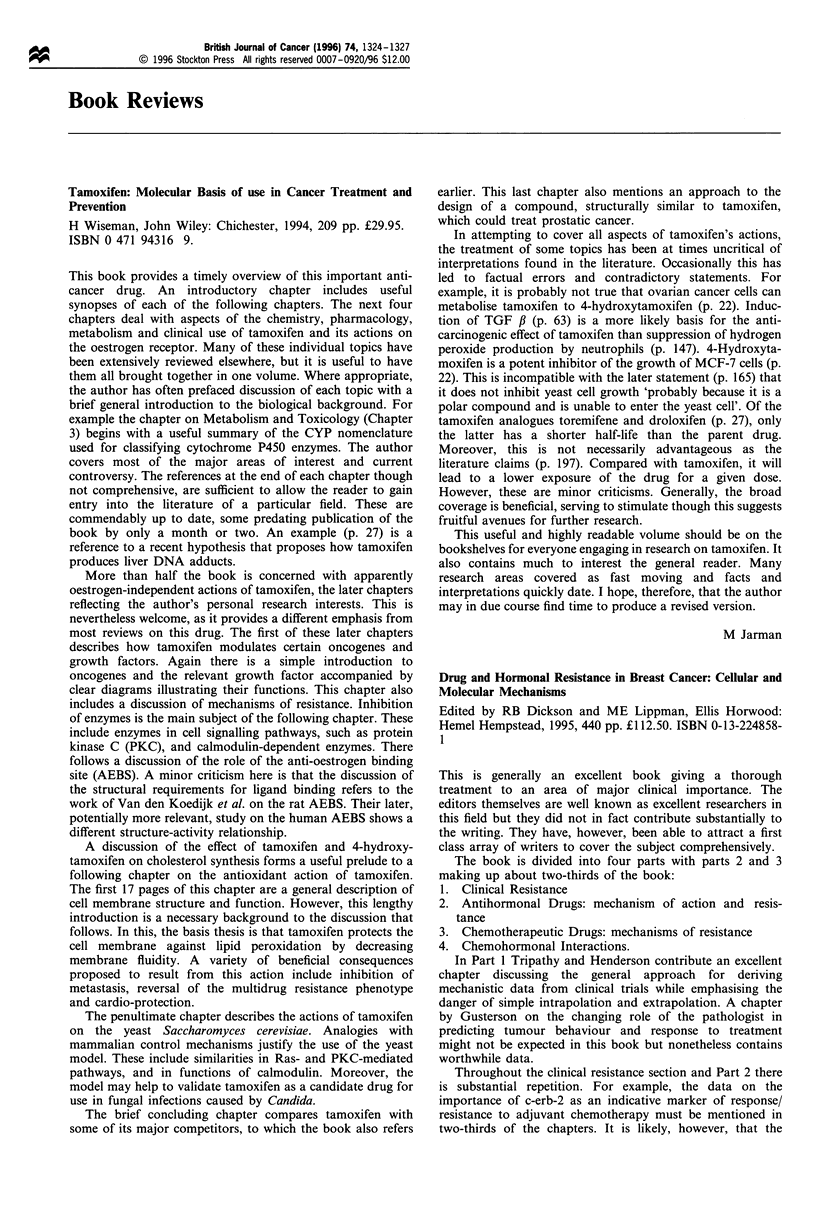# Tamoxifen: molecular basis of use in cancer treatment and prevention

**Published:** 1996-10

**Authors:** M Jarman


© 1996 Stockton Press All rights reserved 0007-0920/96 $12.00

### Book Reviews

**Tamoxifen: Molecular Basis of use in Cancer Treatment and Prevention**

H Wiseman, John Wiley: Chichester, 1994, 209 pp. £29.95. ISBN 0 471 94316 9.

This book provides a timely overview of this important anti-cancer drug. An introductory chapter includes useful synopses of each of the following chapters. The next four chapters deal with aspects of the chemistry, pharmacology, metabolism and clinical use of tamoxifen and its actions on the oestrogen receptor. Many of these individual topics have been extensively reviewed elsewhere, but it is useful to have them all brought together in one volume. Where appropriate, the author has often prefaced discussion of each topic with a brief general introduction to the biological background. For example the chapter on Metabolism and Toxicology (Chapter 3) begins with a useful summary of the CYP nomenclature used for classifying cytochrome P450 enzymes. The author covers most of the major areas of interest and current controversy. The references at the end of each chapter though not comprehensive, are sufficient to allow the reader to gain entry into the literature of a particular field. These are commendably up to date, some predating publication of the book by only a month or two. An example (p. 27) is a reference to a recent hypothesis that proposes how tamoxifen produces liver DNA adducts.

More than half the book is concerned with apparently oestrogen-independent actions of tamoxifen, the later chapters reflecting the author's personal research interests. This is nevertheless welcome, as it provides a different emphasis from most reviews on this drug. The first of these later chapters describes how tamoxifen modulates certain oncogenes and growth factors. Again there is a simple introduction to oncogenes and the relevant growth factor accompanied by clear diagrams illustrating their functions. This chapter also includes a discussion of mechanisms of resistance. Inhibition of enzymes is the main subject of the following chapter. These include enzymes in cell signalling pathways, such as protein kinase C (PKC), and calmodulin-dependent enzymes. There follows a discussion of the role of the anti-oestrogen binding site (AEBS). A minor criticism here is that the discussion of the structural requirements for ligand binding refers to the work of Van den Koedijk *et al.* on the rat AEBS. Their later, potentially more relevant, study on the human AEBS shows a different structure-activity relationship.

A discussion of the effect of tamoxifen and 4-hydroxy-tamoxifen on cholesterol synthesis forms a useful prelude to a following chapter on the antioxidant action of tamoxifen. The first 17 pages of this chapter are a general description of cell membrane structure and function. However, this lengthy introduction is a necessary background to the discussion that follows. In this, the basis thesis is that tamoxifen protects the cell membrane against lipid peroxidation by decreasing membrane fluidity. A variety of beneficial consequences proposed to result from this action include inhibition of metastasis, reversal of the multidrug resistance phenotype and cardio-protection.

The penultimate chapter describes the actions of tamoxifen on the yeast *Saccharomyces cerevisiae.* Analogies with mammalian control mechanisms justify the use of the yeast model. These include similarities in Ras- and PKC-mediated pathways, and in functions of calmodulin. Moreover, the model may help to validate tamoxifen as a candidate drug for use in fungal infections caused by *Candida.*

The brief concluding chapter compares tamoxifen with some of its major competitors, to which the book also refers earlier. This last chapter also mentions an approach to the design of a compound, structurally similar to tamoxifen, which could treat prostatic cancer.

In attempting to cover all aspects of tamoxifen's actions, the treatment of some topics has been at times uncritical of interpretations found in the literature. Occasionally this has led to factual errors and contradictory statements. For example, it is probably not true that ovarian cancer cells can metabolise tamoxifen to 4-hydroxytamoxifen (p. 22). Induction of TGF $\beta$ (p. 63) is a more likely basis for the anti-carcinogenic effect of tamoxifen than suppression of hydrogen peroxide production by neutrophils (p. 147). 4-Hydroxytamoxifen is a potent inhibitor of the growth of MCF-7 cells (p. 22). This is incompatible with the later statement (p. 165) that it does not inhibit yeast cell growth 'probably because it is a polar compound and is unable to enter the yeast cell'. Of the tamoxifen analogues toremifene and droloxifen (p. 27), only the latter has a shorter half-life than the parent drug. Moreover, this is not necessarily advantageous as the literature claims (p. 197). Compared with tamoxifen, it will lead to a lower exposure of the drug for a given dose. However, these are minor criticisms. Generally, the broad coverage is beneficial, serving to stimulate though this suggests fruitful avenues for further research.

This useful and highly readable volume should be on the bookshelves for everyone engaging in research on tamoxifen. It also contains much to interest the general reader. Many research areas covered as fast moving and facts and interpretations quickly date. I hope, therefore, that the author may in due course find time to produce a revised version.

M Jarman